# Sleep duration and its association with constipation in patients with diabetes: The fukuoka diabetes registry

**DOI:** 10.1371/journal.pone.0302430

**Published:** 2024-05-22

**Authors:** Toshiaki Ohkuma, Masanori Iwase, Takanari Kitazono

**Affiliations:** 1 Department of Medicine and Clinical Science, Graduate School of Medical Sciences, Kyushu University, Fukuoka, Japan; 2 Diabetes Center, Hakujyuji Hospital, Fukuoka, Japan; Juntendo University: Juntendo Daigaku, JAPAN

## Abstract

**Aims:**

Shorter and longer sleep durations are associated with adverse health consequences. However, available evidence on the association of sleep duration with constipation is limited, especially in patients with diabetes, who are at a high risk of both conditions. This study aimed to examine the association between sleep duration and constipation in patients with type 2 diabetes.

**Methods:**

A total of 4,826 patients with type 2 diabetes were classified into six groups according to sleep duration: <4.5, 4.5–5.4, 5.5–6.4, 6.5–7.4, 7.5–8.4, and ≥8.5 hours/day. The odds ratios for the presence of constipation, defined as a defecation frequency <3 times/week and/or laxative use, were calculated using a logistic regression model.

**Results:**

Shorter and longer sleep durations were associated with a higher likelihood of constipation than an intermediate duration (6.5–7.4 hours/day). This U-shaped association persisted after adjusting for confounding factors, including lifestyle behavior, measures of obesity and glycemic control, and comorbidities. Broadly identical findings were observed when decreased defecation frequency and laxative use were individually assessed.

**Conclusions:**

This study shows a U-shaped association between sleep duration and constipation in patients with type 2 diabetes, and highlights the importance of assessing sleep duration in daily clinical practice.

## Introduction

Habitual sleep durations have decreased as lifestyles have changed in recent decades. The National Health Interview Survey showed that, between 1985 and 2012, mean sleep duration decreased (7.40 to 7.18 hours), and the percentage of adults sleeping ≤6 hours increased (22.3% to 29.2%) [1]. In accordance with this decrease in sleep duration, a growing number of epidemiological studies have reported that a shorter sleep duration is associated with an increased risk of adverse health consequences, such as premature death [2], CVD [3], and their related risk factors of diabetes [4], obesity [5], and hypertension [6]. A longer sleep duration is also related to an elevated risk of these health outcomes [2–4, 6, 7]. These findings indicate the existence of U-shaped relationship between sleep duration and adverse health outcomes, and therefore having adequate amount of sleep is recommended to improve health status [8].

Constipation, which is one of the most common gut disorders, has recently been reported to be a risk factor for premature death [9] and CVD [9–11]. Constipation is also associated with a higher likelihood of diabetes [12, 13], and is the most frequent gastrointestinal symptom in patients with diabetes. In addition, associations of constipation with hyperglycemia and chronic kidney disease have been reported in these patients [14, 15]. In conjunction with constipation-related decrease in quality of life, the importance of prevention and management of constipation has also been widely recognized. With regard to the relationship between constipation and sleep duration, a recent study showed a possible association between shorter and longer sleep durations and constipation [16]. However, to date, no study has clarified this issue in patients with diabetes. Taking into account the fact that patients with diabetes have a high risk of sleep-related problems and constipation, the association between these conditions may differ among patients with diabetes. Furthermore, because of the above-mentioned associations of sleep duration [3] and constipation [9–11] with the risk of CVD, clarifying the association between these factors may be helpful in reducing the long-term risk of CVD.

Therefore, this study aimed to examine the association between sleep duration and constipation in patients with type 2 diabetes.

## Subjects and methods

### Study design and population

The Fukuoka Diabetes Registry is a multicenter, prospective cohort study designed to investigate the effect of modern treatments on the prognoses of patients with diabetes who regularly attend teaching hospitals (UMIN Clinical Trial Registry 000002627). These hospitals are certified by the Japan Diabetes Society or certified diabetes clinics in Fukuoka Prefecture, Japan [17]. In brief, 5,131 patients with diabetes aged ≥20 years were recruited between April 2008 and October 2010. The exclusion criteria of this registry were as follows: 1) patients with drug-induced diabetes or undergoing steroid treatment; 2) patients under renal replacement therapy; 3) patients with serious diseases other than diabetes, such as advanced malignancies, and decompensated liver cirrhosis; and 4) patients unable to visit diabetologists regularly. After excluding 208 participants with type 1 diabetes (negative serum C-peptide under insulin treatment), 6 with missing information on defecation frequency, and 91 with a history of colon cancer, the remaining 4,826 participants with type 2 diabetes were included in the present cross-sectional study. This study was conducted with the approval of the Kyushu University Institutional Review Board, and written informed consent was obtained from all participants.

### Clinical evaluation and laboratory measurements

Participants completed a questionnaire covering their sleep duration, defecation frequency, laxative use, duration of diabetes, diet, smoking and alcohol drinking habits, leisure time physical activity (LTPA), depressive symptoms, presence of dysesthesia in both feet, and a history of retinal photocoagulation and CVD (coronary heart disease or stroke). Sleep duration was self-reported and assessed by the answer to the question, “How long is your habitual sleep duration, including naps?” [18]. The participants were divided into six groups according to their sleep duration: <4.5, 4.5–5.4, 5.5–6.4, 6.5–7.4, 7.5–8.4, and ≥8.5 hours/day. Dietary habits were surveyed by a brief-type self-administered diet history questionnaire regarding the food frequency of 58 items (BDHQ; Gender Medical Research Inc., Tokyo, Japan). The validity of ranking the energy-adjusted intakes of dietary fiber in this questionnaire was previously examined in an adult Japanese population [19]. LTPA was assessed by a self-administered questionnaire, and metabolic equivalent hours per week (MET·h/w) were computed [20]. Depressive symptoms were evaluated using the Center for Epidemiologic Studies Depression Scale [21], and participants who scored ≥16 of 60 points were defined as having depressive symptoms. The participants were categorized as taking or not taking oral hypoglycemic agents or insulin therapy. BMI was calculated from height and weight. Blood pressure was measured with the participant in the sitting position. Collection of blood was performed by venipuncture. HbA_1c_ was determined by high-performance liquid chromatography (Tosoh Corp., Tokyo, Japan) and serum creatinine by enzymatic methods. The estimated glomerular filtration rate was calculated by the equation proposed by the Japanese Society of Nephrology [22], and decreased kidney function was defined as an estimated glomerular filtration rate <60 ml/min/1.73 m^2^.

### Study outcomes

The primary outcome of the present study was the presence of constipation. Constipation was defined as a defecation frequency <3 times/week and/or laxative use, which are the major symptoms of constipation in the Rome IV criteria [23]. Secondary outcomes were the individual components of the primary outcome, namely, decreased defecation frequency and laxative use.

### Statistical analysis

Differences in the mean values or proportions of the characteristics of the study participants were tested using analysis of variance or the chi-square test, as appropriate. The proportions of participants with constipation and related components were adjusted for age and sex by a direct method using all study participants as a standard population, and they were compared using logistic regression analysis. Multivariable-adjusted odds ratios and their 95% CIs for the outcomes were calculated using a logistic regression model. The models were adjusted for age, sex, duration of diabetes, current smoking, current alcohol drinking habit, total dietary fiber intake, LTPA, BMI, HbA_1c_, biguanide use, and insulin use. The associations between sleep duration as a continuous variable and the outcomes were also examined using restricted cubic spline regression models with knots placed at sleep durations of 4.5, 5.5, 6.5, 7.5, and 8.5 hours/day. The data were accessed on May 1, 2023. All analyses were performed using the SAS software package version 9.4 (SAS Institute Inc., Cary, NC) and Stata software (release 16.1, StataCorp, College Station, TX). Values of *p<*0.05 were considered to be statistically significant in all analyses.

## Results

The clinical characteristics of the study participants according to sleep duration are shown in Table 1. Participants with a shorter sleep duration were more likely to be younger, women, have a shorter duration of diabetes, and have a higher BMI. HbA_1c_ values were higher in participants with shorter and longer sleep durations than an intermediate duration. The proportion of participants treated with oral hypoglycemic agents was not significantly different across the groups, except for biguanides. The proportion of participants on biguanide medication was higher in those with a shorter sleep duration than in those with a longer sleep duration. The proportion of insulin use was likely to be higher in association with an increase in sleep duration. Regarding comorbidities, shorter and longer sleep durations were associated with a higher likelihood of having dysesthesia in both feet and a history of retinal photocoagulation. The proportion of participants with decreased kidney function and a history of CVD tended to be higher in those with a longer sleep duration, while that of participants with depressive symptoms was higher in those with a shorter sleep duration.

**Table 1:** Clinical characteristics of the participants according to sleep duration.

	Sleep duration (hours/day)						
	<4.5	4.5–5.4	5.5–6.4	6.5–7.4	7.5–8.4	≥8.5	P value
n	163	536	1,217	1,330	1,122	458	
Age (years)	62.8 (11.3)	63.6 (10.7)	63.4 (10.1)	64.4 (10.2)	67.6 (9.4)	70.3 (9.2)	<0.001
Women (%)	57.1% (93)	52.6% (282)	45.4% (552)	43.8% (582)	39.8% (446)	34.5% (158)	<0.001
Duration of diabetes mellitus (years)	14.8 (10.4)	14.3 (10.4)	14.4 (9.9)	14.9 (10.1)	17.3 (11.1)	17.5 (11.1)	<0.001
Current smoking habit (%)	23.3% (38)	16.8% (90)	20.5% (249)	18.5% (246)	16.8% (188)	17.7% (81)	0.10
Current alcohol drinking (%)	28.2% (46)	37.9% (203)	41.2% (501)	38.6% (513)	38.9% (436)	38.2% (175)	0.055
Total energy intake (kcal/day)	1632 (541)	1637 (518)	1685 (469)	1703 (501)	1695 (487)	1719 (520)	0.07
Dietary fiber intake (g/1,000kcal)	7.7 (2.1)	7.6 (2.4)	7.5 (2.2)	7.7 (2.3)	7.5 (2.1)	7.6 (2.2)	<0.001
LTPA (MET·h/w)	10.2 (14.8)	10.2 (15.7)	10.8 (14.3)	11.7 (14.1)	13.1 (15.7)	12.2 (15.6)	<0.001
BMI (kg/m^2^)	25.3 (4.4)	24.7 (4.2)	23.9 (3.8)	23.6 (3.8)	23.4 (3.4)	23.4 (3.5)	<0.001
HbA_1c_ (%)	7.71 (1.18)	7.53 (1.11)	7.44 (1.02)	7.39 (1.05)	7.40 (1.00)	7.44 (1.05)	0.002
Systolic BP (mmHg)	130.3 (16.3)	131.5 (17.5)	129.9 (16.8)	130.1 (16.4)	130.6 (17.6)	132.1 (18.3)	0.15
Diastolic BP (mmHg)	73.9 (10.2)	75.2 (10.3)	74.9 (10.5)	74.5 (10.6)	74.0 (10.7)	73.5 (11.3)	0.03
Oral hypoglycemic agent use (%)	69.3% (113)	63.1% (338)	64.8% (789)	62.3% (828)	64.2% (720)	60.3% (276)	0.26
Biguanides (%)	37.4% (61)	35.6% (191)	37.1% (452)	33.5% (445)	29.8% (334)	26.6% (122)	<0.001
Sulfonylureas (%)	48.5% (79)	40.9% (219)	42.2% (514)	40.2% (534)	45.0% (505)	40.4% (185)	0.09
Thiazolidinediones (%)	16.0% (26)	12.7% (68)	13.2% (161)	12.9% (172)	12.8% (143)	12.5% (57)	0.91
α-Glucosidase inhibitors	9.2% (15)	10.1% (54)	10.7% (130)	12.0% (160)	12.8% (143)	11.1% (51)	0.43
Glinides	5.5% (9)	5.0% (27)	5.4% (66)	5.7% (76)	6.1% (68)	5.5% (25)	0.97
DPP-4 inhibitors	1.2% (2)	0.6% (3)	0.3% (4)	0.3% (4)	0.5% (5)	0.2% (1)	0.53
GLP-1 receptor agonists	0% (0)	0.2% (1)	0% (0)	0.1% (1)	0.2% (2)	0% (0)	0.63
Insulin use (%)	27.0% (44)	28.5% (153)	26.5% (322)	28.7% (382)	29.1% (326)	34.5% (158)	0.055
Dysesthesia of both feet (%)	33.7% (55)	20.2% (108)	18.5% (225)	17.1% (228)	20.7% (232)	23.4% (107)	<0.001
History of retinal photocoagulation (%)	31.3% (51)	19.2% (103)	19.3% (235)	18.7% (249)	24.0% (269)	29.0% (133)	<0.001
Decreased kidney function (%)	20.3% (33)	16.2% (87)	15.9% (194)	18.4% (244)	26.3% (295)	34.7% (159)	<0.001
eGFR (mL/min/1.73 m^2^)	77.4 (25.2)	77.2 (21.6)	77.9 (20.5)	76.0 (21.0)	72.3 (21.5)	67.0 (23.7)	<0.001
History of cardiovascular disease (%)	20.9% (34)	21.1% (113)	18.3% (223)	19.3% (257)	25.0% (281)	31.2% (143)	<0.001
Depressive symptoms (%)	25.8% (42)	13.4% (72)	7.5% (91)	7.1% (94)	7.8% (87)	9.2% (42)	<0.001

Values shown as the mean (standard deviation) for continuous variables, and as the percentage (number) for categorical variables.

Abbreviations: DPP-4, dipeptidyl peptidase 4; eGFR, estimated glomerular filtration rate; GLP-1, glucagon-like peptide 1; LTPA, leisure time physical activity; MET h/w, metabolic equivalent hours per week.

**Fig 1:**
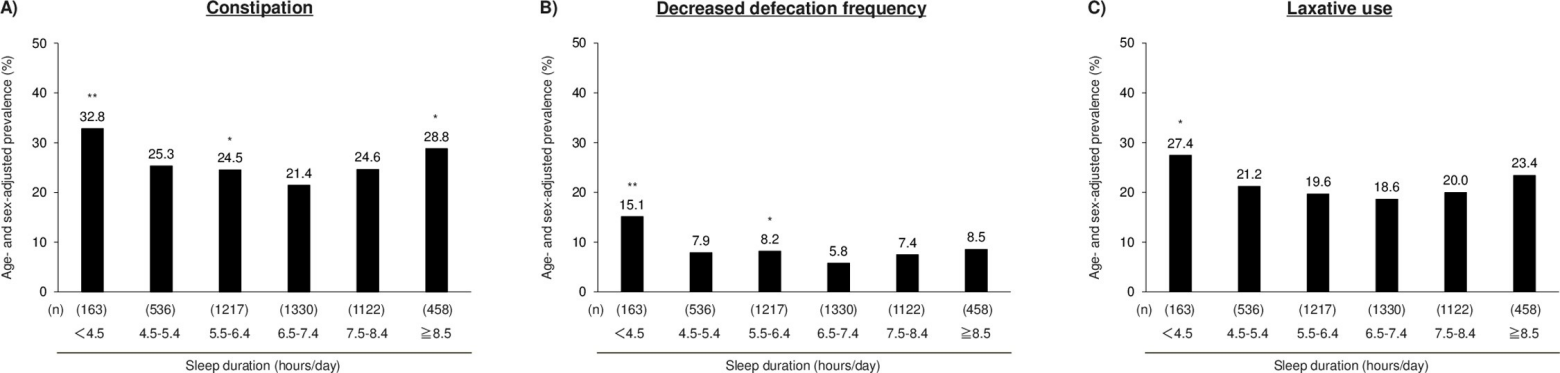
Age- and sex-adjusted prevalence of constipation and related components according to sleep duration. Decreased defecation frequency was defined as <3 times/week. *p<0.05, **p<0.001 vs. a sleep duration of 6.5–7.4 hours/day.

The age- and sex-adjusted prevalence of constipation is shown in Fig 1. Participants with shorter and longer sleep durations showed a higher prevalence of constipation than those who slept for 6.5–7.4 hours. The prevalence of constipation was 32.8%, 25.3%, 24.5%, 21.4%, 24.6%, and 28.8% in participants with a sleep duration of <4.5, 4.5–5.4, 5.5–6.4, 6.5–7.4, 7.5–8.4, and ≥8.5 hours/day (Fig 1A), which indicated a U-shaped association. Similar U-shaped associations were also observed for the related components of constipation, namely, the defecation frequency (Fig 1B) and laxative use (Fig 1C).

**Fig 2:**
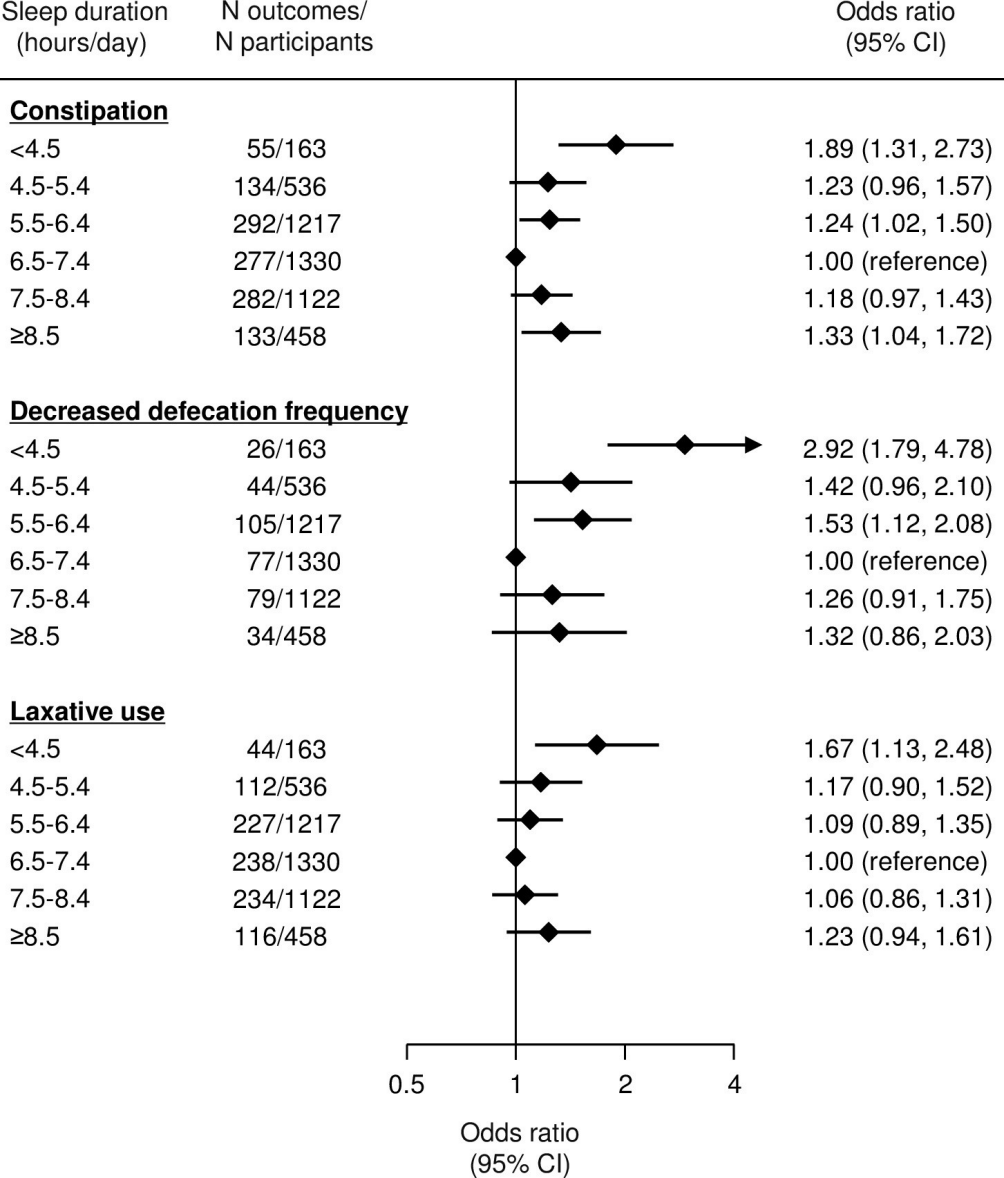
Multivariable-adjusted odds ratios and 95% CIs for constipation and related components according to sleep duration. Decreased defecation frequency was defined as <3 times/week. Models were adjusted for age, sex, duration of diabetes, current smoking, current alcohol drinking habit, total dietary fiber intake, leisure time physical activity, BMI, HbA_1c_, biguanide use, and insulin use. Data in the graphs represent odds ratios and 95% CIs.

**Fig 3:**
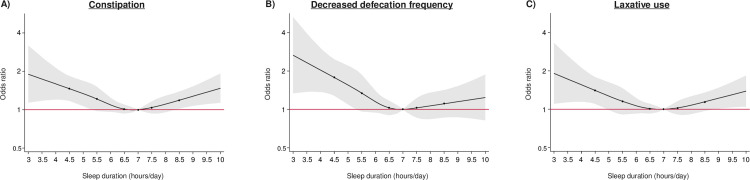
Spline curves for assessing the association between sleep duration and constipation and related components. Decreased defecation frequency was defined as <3 times/week. The circles at 4.5, 5.5, 6.5, 7.5, and 8.5 hours/day represent the points at which knots were placed. The areas shaded in gray represent the 95% CIs. The red lines represent odds ratios of 1. Values were trimmed at >10 hours/day (0.68% of the participants). Models were adjusted for age, sex, duration of diabetes, current smoking, current alcohol drinking habit, total dietary fiber intake, leisure time physical activity, BMI, HbA_1c_, biguanide use, and insulin use.

Fig 2 shows the multivariable-adjusted ORs for constipation according to sleep duration. Participants with a shorter or longer sleep duration were more likely to have constipation than those with a sleep duration of 6.5–7.4 hours. The ORs for constipation were 1.89 (95% CI 1.31, 2.73) for <4.5 hours, 1.23 (0.96, 1.57) for 4.5–5.4 hours, 1.24 (1.02, 1.50) for 5.5–6.4 hours, 1.00 (reference) for 6.5–7.4 hours, 1.18 (0.97, 1.43) for 7.5–8.4 hours, and 1.33 (1.04, 1.72) for ≥8.5 hours. Additional adjustments for comorbidities, such as the presence of dysesthesia in both feet, a history of retinal photocoagulation, decreased kidney function, a history of CVD, and the presence of depressive symptoms, did not substantially alter this association (S1 Fig). Subgroup analyses by sex also showed a similar association (S2 Fig). Broadly identical findings were found for the ORs of decreased defecation frequency and laxative use (Fig 2). Multivariable-adjusted ORs were 2.92 (1.79, 4.78) for a decreased defecation frequency and 1.67 (1.13, 2.48) for laxative use in participants with a sleep duration of <4.5 hours compared with 6.5–7.4 hours. A similar elevated likelihood was observed for a longer sleep duration, and the corresponding ORs for ≥8.5 hours were 1.32 (0.86, 2.03) for a decreased defecation frequency and 1.23 (0.94, 1.61) for laxative use. The magnitude of these associations was almost identical to that of constipation (1.33 [1.04, 1.72]), but they did not reach statistical significance. Furthermore, mutual adjustment for the component of constipation (i.e. adjusting for decreased defecation frequency for the analysis of laxative use, and vice versa) did not materially alter the U-shaped association (S3 Fig). When sleep duration was assessed as a continuous variable instead of a categorical variable, similar U-shaped associations were found for constipation, as well as its components of decreased defecation frequency and laxative use individually. A sleep duration of 7 hours showed the lowest risk (Fig 3).

## Discussion

This study showed that shorter and longer sleep durations were significantly associated with a higher likelihood of having constipation compared with an intermediate sleep duration in patients with type 2 diabetes. The U-shaped association persisted after adjustment for confounding factors, including lifestyle behavior, measures of obesity and glycemic control, and comorbidities. Furthermore, this association was similar for decreased defecation frequency and laxative use individually. To the best of our knowledge, the present study is the first to show a U-shaped association between sleep duration and constipation in patients with diabetes, who are at high risk of sleep problems and constipation. These findings suggest the importance of assessing sleep duration in diabetes clinical practice to avoid constipation and to decrease the long-term risk of CVD.

Accumulating epidemiological studies have shown an association between sleep duration and health outcomes, such as mortality [2] and CVD [3], as well as their risk factors such as diabetes [4], obesity [5, 7], and hypertension [6]. In these studies, an elevated risk of these outcomes was found with shorter and longer sleep durations. However, studies that have reported an association between sleep duration and gastrointestinal disorders are limited. Constipation is one of the major gastrointestinal symptoms and has recently attracted attention as a risk factor for CVD [9–11]. A cross-sectional analysis from NHANES showed that shorter and longer sleep durations were associated with a higher likelihood of constipation [16], which was defined on the basis of stool form (Bristol Stool Form Scale type) or laxative use. Compared with a sleep duration of 7 hours, a shorter sleep duration was significantly associated with constipation (OR: 1.25 [95% CI 1.03–1.51] for 6 hours, 1.57 [1.25–1.98] for 5 hours, and 1.54 [1.16–2.04] for ≤4 hours). The corresponding ORs for a longer sleep duration were 1.16 (0.96–1.39) for 8 hours, 1.05 (0.76–1.46) for 9 hours, and 1.90 (1.33–2.72) for ≥10 hours. Other studies on college freshmen [24] or elementary school students [25] also assessed this issue, but showed inconsistent results of a null [24] or positive [25] association between a shorter sleep duration and constipation. However, the available evidence on this topic in the adult population is limited. The present study showed a U-shaped association between sleep duration and laxative use, and this association was also observed between sleep duration and decreased defecation frequency, which is another important component of constipation. In addition, although previous studies on this topic were conducted in the general population, to the best of our knowledge, this is the first study to show a U-shaped association between sleep duration and constipation in patients with diabetes. Patients with diabetes are at increased risk of sleep problems [26] and constipation [13]. Taken together with findings of a close association of constipation with mortality [9], CVD [9–11], and chronic kidney disease [15, 27], the current findings suggest the usefulness of assessing sleep duration to decrease long-term clinical outcomes.

There are several potential mechanisms underlying the association between sleep duration and constipation. Sleep deprivation is associated with changes in the gut microbiota, as shown in human and animal studies [28–30]. Sleep deprivation may consequently lead to constipation because of the association between changes in intestinal microflora and constipation [31–33]. Another possible mechanism may be activation of the sympathetic nervous system following a short sleep duration [34]. The activation of sympathetic nerves may increase the risk of constipation via motor inhibition of the small and large intestines [35]. In addition, lifestyle and psychological factors, such as decreased physical activity and a higher prevalence of depressive symptoms (Table 1), may contribute to the association between a shorter sleep duration and constipation. These factors are also associated with a longer sleep duration [36], and may also confound the association of constipation with a longer sleep duration. However, a significant association persisted after adjustment for these factors in our study, which suggests that other mechanisms are involved. With regard to the association of constipation with a longer sleep duration, sleep-disordered breathing, which is highly prevalent in patients with diabetes [37], may be involved in this association. Sleep fragmentation and intermittent hypoxia, which are the major characteristics of sleep-disordered breathing, are associated with alterations in the gut microbiota [38], and these may subsequently result in constipation.

A strength of the current study is that we had a study population with a relatively large number of patients with type 2 diabetes because of the emerging prevalence of diabetes and an elevated risk of sleep and gastrointestinal disorders associated with diabetes. Furthermore, assessment of the defecation frequency allowed us to examine the association of sleep duration with different aspects of constipation, in addition to the stool form or laxative use, which was assessed in a previous study [16]. Additionally, a comprehensive multivariable adjustment for confounders, such as lifestyle factors of dietary fiber intake, exercise, and smoking, as well as comorbidities including depressive symptoms, was conducted to determine the association between sleep duration and constipation. However, some limitations should be mentioned. First, sleep duration was assessed by a self-reported questionnaire and was not objectively evaluated. However, self-reported sleep duration was used in many previous epidemiological studies, and the validation studies showed a moderate correlation with objective measured sleep duration by wrist actigraphy (r = 0.45–0.57) [39, 40]. Second, we evaluated the quantity of sleep, but not the quality of sleep, such as proportion of REM and non-REM sleep. REM sleep duration was shown to be associated with gut microbiota composition [41], and may be related to constipation. In addition, the contribution of sleep disorders, such as sleep-disordered breathing, to the observed association was not clarified. Fourth, other factors of constipation, such as straining and sensation of incomplete evacuation, were not evaluated in this study. Fifth, we cannot infer any cause-and-effect associations because of the cross-sectional study design. Finally, there may have been residual confounding factors other than those included in the present study.

In conclusion, this study shows that there is a U-shaped association between sleep duration and constipation in patients with type 2 diabetes. There are close associations of sleep duration and constipation with the long-term risk of CVD and mortality. Therefore, the current findings highlight the need for assessing sleep duration in the management of diabetes to improve long-term prognoses.

## Supporting information

S1 Checklist (PDF)

S1 Fig Sensitivity analyses: multivariable-adjusted odds ratios and 95% CIs for constipation according to sleep duration. Base models were adjusted for age, duration of diabetes, current smoking, current alcohol drinking habit, total dietary fiber intake, leisure time physical activity, BMI, HbA_1c_, biguanide use, and insulin use. Data in the graphs represent odds ratios and 95% CIs. CVD, cardiovascular disease; hx; history.
(PDF)

S2 Fig Subgroup analyses: multivariable-adjusted odds ratios and 95% CIs for constipation according to sleep duration and sex. Decreased defecation frequency was defined as <3 times/week. Models were adjusted for age, duration of diabetes, current smoking, current alcohol drinking habit, total dietary fiber intake, leisure time physical activity, BMI, HbA_1c_, biguanide use, and insulin use. Data in the graphs represent odds ratios and 95% CIs.
(PDF)

S3 Fig Sensitivity analyses: multivariable-adjusted odds ratios and 95% CIs for components of constipation according to sleep duration. Decreased defecation frequency was defined as <3 times/week. Base models were adjusted for age, duration of diabetes, current smoking, current alcohol drinking habit, total dietary fiber intake, leisure time physical activity, BMI, HbA_1c_, biguanide use, and insulin use. Model for decreased defecation frequency was adjusted for the variables in base model plus laxative use. Model for laxative use was adjusted for the variables in base model plus decreased defecation frequency. Data in the graphs represent odds ratios and 95% CIs.
(PDF)
