# Supplementary figures and images for: Sleep duration and its association with constipation in patients with diabetes: The fukuoka diabetes registry
Source: PLoS One. 2024 May 22;19(5):e0302430. doi: 10.1371/journal.pone.0302430 (PMC11111002; doi:10.1371/journal.pone.0302430)

**S1 Fig**

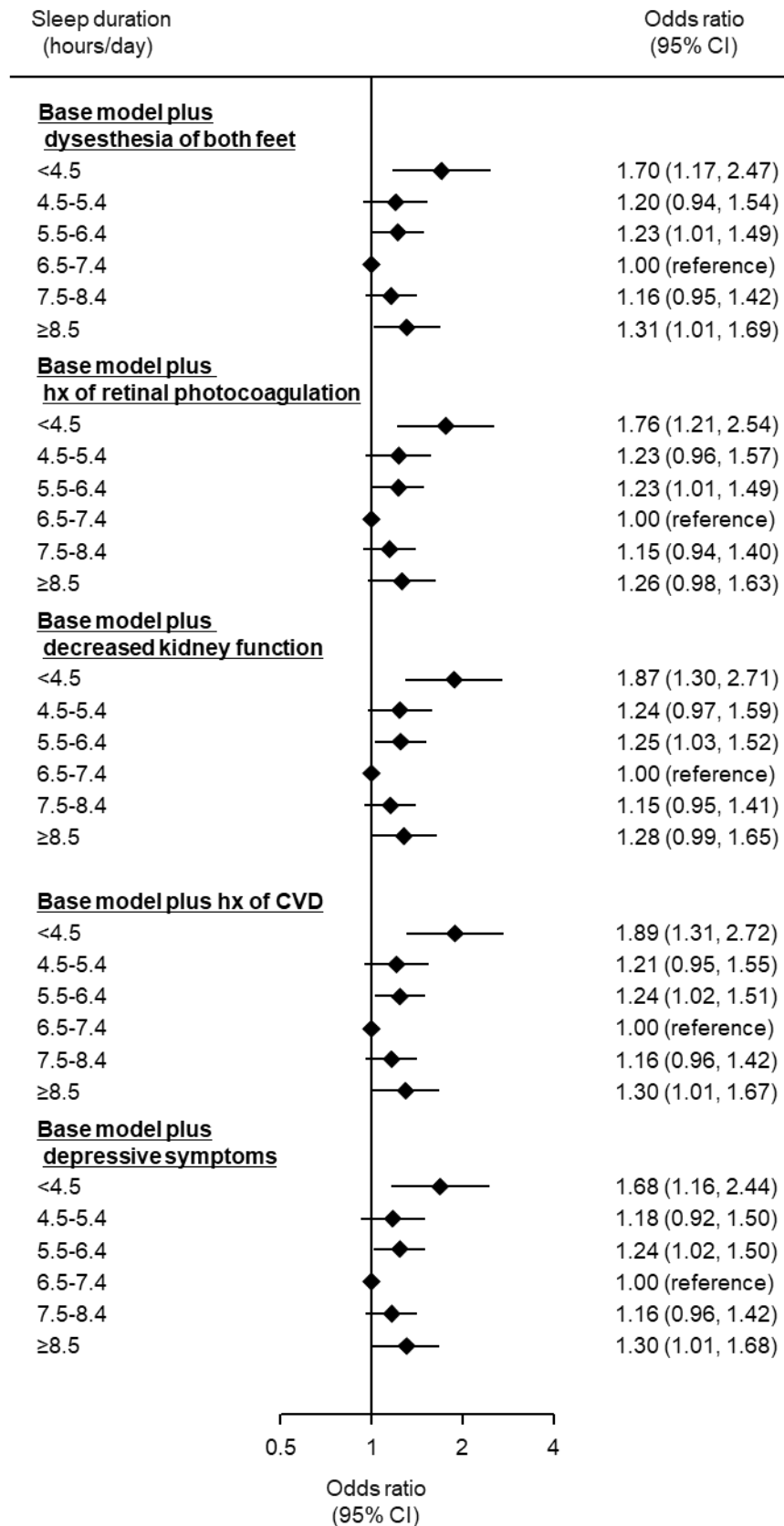

Supplement: S1 Fig — Base models were adjusted for age, duration of diabetes, current smoking, current alcohol drinking habit, total dietary fiber intake, leisure time physical activity, BMI, HbA1c, biguanide use, and insulin use. Data in the graphs represent odds ratios and 95% CIs. CVD, cardiovascular disease; hx; history. (PDF) [file pone.0302430.s002.pdf]

**S2 Fig**

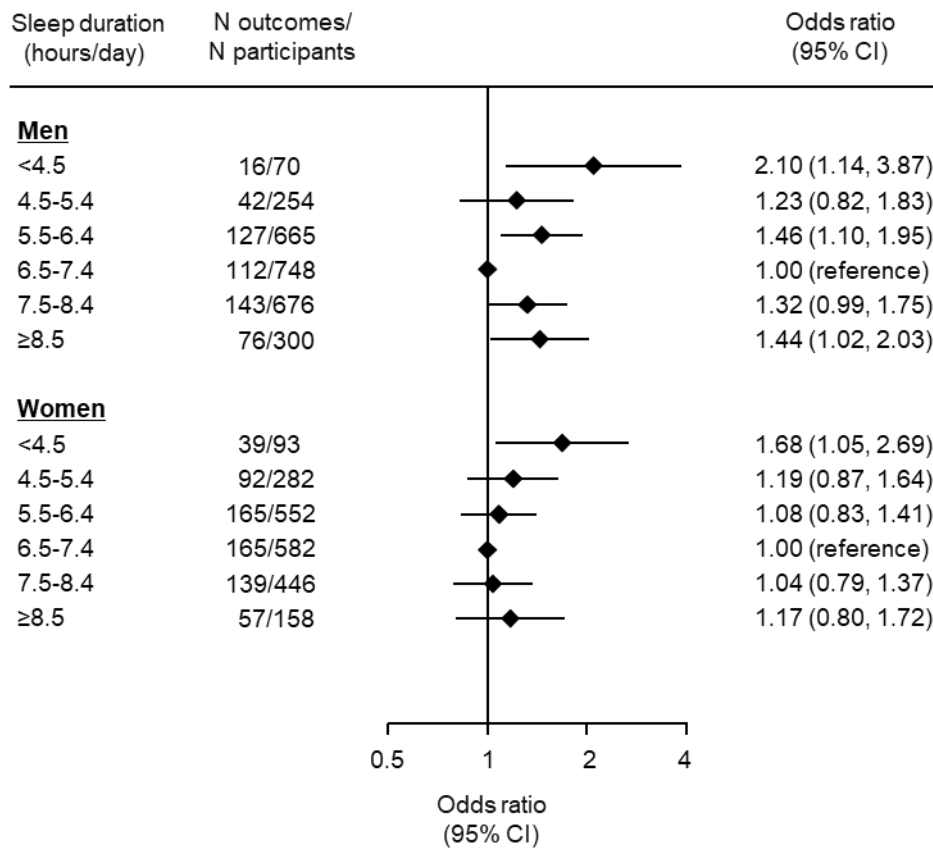

Supplement: S2 Fig — Decreased defecation frequency was defined as <3 times/week. Models were adjusted for age, duration of diabetes, current smoking, current alcohol drinking habit, total dietary fiber intake, leisure time physical activity, BMI, HbA1c, biguanide use, and insulin use. Data in the graphs represent odds ratios and 95% CIs. (PDF) [file pone.0302430.s003.pdf]

S3 Fig

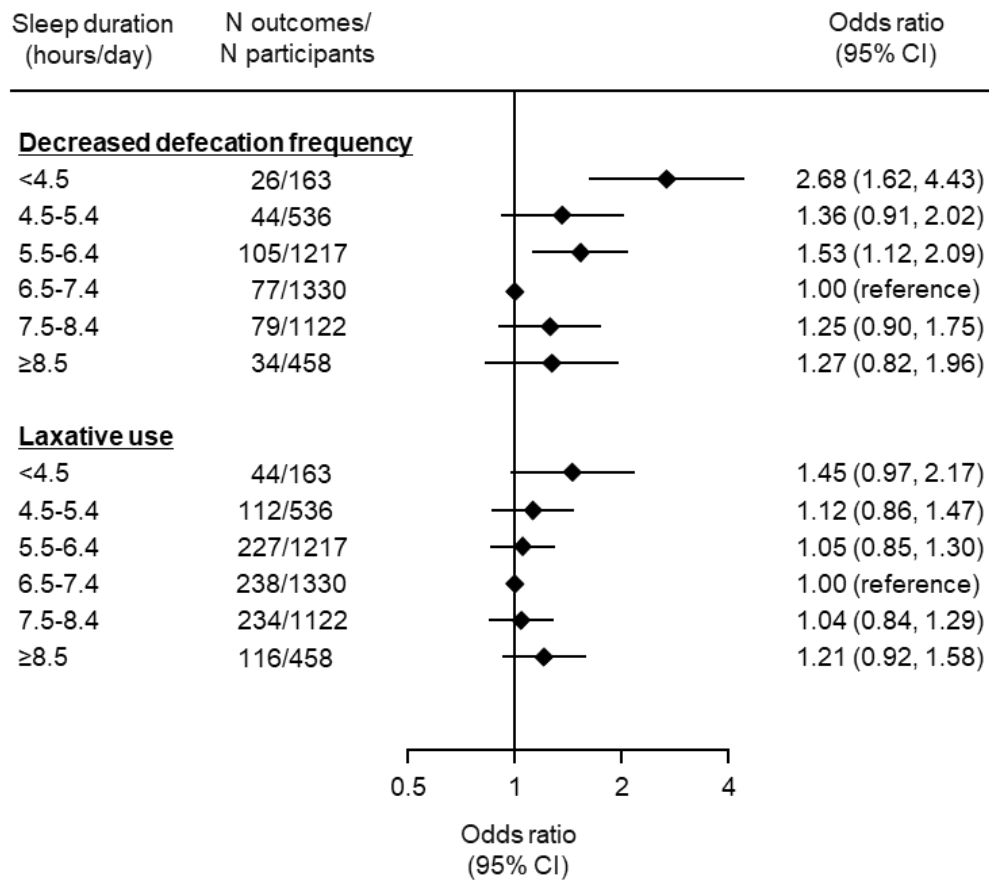

Supplement: S3 Fig — Decreased defecation frequency was defined as <3 times/week. Base models were adjusted for age, duration of diabetes, current smoking, current alcohol drinking habit, total dietary fiber intake, leisure time physical activity, BMI, HbA1c, biguanide use, and insulin use. Model for decreased defecation frequency was adjusted for the variables in base model plus laxative use. Model for laxative use was adjusted for the variables in base model plus decreased defecation frequency. Data in the graphs represent odds ratios and 95% CIs. (PDF) [file pone.0302430.s004.pdf]
